# In Vivo Sustained Release of the Retrograde Transport Inhibitor Retro-2.1 Formulated in a Thermosensitive Hydrogel

**DOI:** 10.3390/ijms232314611

**Published:** 2022-11-23

**Authors:** Robin Vinck, Laetitia Anvi Nguyen, Mathilde Munier, Lucie Caramelle, Diana Karpman, Julien Barbier, Alain Pruvost, Jean-Christophe Cintrat, Daniel Gillet

**Affiliations:** 1 SIMoS, Département Médicaments et Technologies pour la Santé (DMTS), Université Paris-Saclay, CEA, INRAE, 91191 Gif-sur-Yvette, France; 2 SCBM, Département Médicaments et Technologies pour la Santé (DMTS), Université Paris-Saclay, CEA, INRAE, 91191 Gif-sur-Yvette, France; 3 SPI, Département Médicaments et Technologies pour la Santé (DMTS), Université Paris-Saclay, CEA, INRAE, 91191 Gif-sur-Yvette, France; 4 Department of Pediatrics, Clinical Sciences Lund, Lund University, 22242 Lund, Sweden

**Keywords:** retrograde transport inhibitor, broad spectrum, Retro-2.1, formulation, thermosensitive hydrogel, pharmacokinetic, metabolism

## Abstract

A recently developed inhibitor of retrograde transport, namely Retro-2.1, proved to be a potent and broad-spectrum lead in vitro against intracellular pathogens, such as toxins, parasites, intracellular bacteria and viruses. To circumvent its low aqueous solubility, a formulation in poly(ethylene glycol)-*block*-poly(D,L)lactide micelle nanoparticles was developed. This formulation enabled the study of the pharmacokinetic parameters of Retro-2.1 in mice following intravenous and intraperitoneal injections, revealing a short blood circulation time, with an elimination half-life of 5 and 6.7 h, respectively. To explain the poor pharmacokinetic parameters, the metabolic stability of Retro-2.1 was studied in vitro and in vivo, revealing fast cytochrome-P-450-mediated metabolism into a less potent hydroxylated analogue. Subcutaneous injection of Retro-2.1 formulated in a biocompatible and bioresorbable polymer-based thermosensitive hydrogel allowed for sustained release of the drug, with an elimination half-life of 19 h, and better control of its metabolism. This study provides a guideline on how to administer this promising lead in vivo in order to study its efficacy.

## 1. Introduction

**Figure 1:**
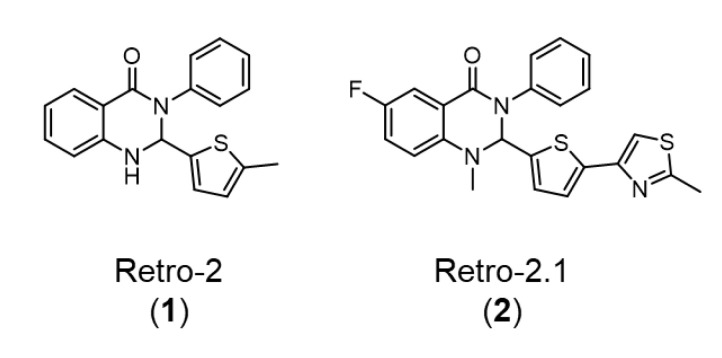
Structures of Retro-2 (**1**) and its second-generation derivative Retro-2.1 (**2**).

2-(((5-Methylthiophen-2-yl)methylene)amino)-N-phenylbenzamide (**1**), also called Retro-2 (Figure 1), was first identified during a phenotypic high-throughput screening campaign as an inhibitor of ricin, a highly potent plant toxin. The study of its mode of action revealed that Retro-2 is able to inhibit the retrograde transport of ricin to the endoplasmic reticulum, preventing its eventual translocation to the cytoplasm. Thanks to this unique mode of action, Retro-2 has the potential to act as a broad-spectrum drug candidate towards any pathogen exploiting retrograde transport to replicate or reach its intracellular target [1].

To further improve the potency of Retro-2, a structure–activity relationship study was performed and led to the discovery of a more efficient version of the retrograde pathway inhibitor, 6-fluoro-1-methyl-2-(5-(2-methylthiazol-4-yl)thiophen-2-yl)-3-phenyl-2,3-dihydroquinazolin-4(1H)-1, later named Retro-2.1 (Figure 1). Of note, the (*S*)-enantiomer of Retro-2.1 demonstrated the highest potency, with an EC_50_ value of 54 nM against the Shiga toxin [2,3].

Over the years, Retro-2 and Retro-2.1 demonstrated the ability to inhibit the effect or proliferation of intracellular pathogens, such as toxins, parasites, intracellular bacteria and viruses [1,2,3,4,5,6,7,8,9,10,11,12,13,14,15,16]. This list of pathogens is still growing, with the recent addition of SARS-CoV-2, demonstrating the high therapeutical potential of the Retro-2 series (Table 1) [17]. Moreover, the recent unravelling of its intracellular target unlocked the potential discovery of new applications [18]. Only a few of the tested pathogens were found to be unsensitive to the Retro-2 family (i.e., diphtheria toxin, *Clostridium botulinum* neurotoxin A, dengue virus serotype 4, chikungunya virus and Venezuelan equine encephalitis virus) [4].

Due to this wide variety of pathogens, and the differences in their targeted organs, Retro-2.1 would greatly benefit from a pharmaceutical formulation and mode of administration favouring systemic exposition. This would allow a study of Retro-2.1 in the corresponding in vivo models and would give a starting point towards the development of a clinical formulation. However, Retro-2.1 suffers from low aqueous solubility (below 1 mg/L), preventing its parenteral administration. Attempts at increasing its hydrophilicity through the introduction of hydrophilic moieties has so far failed at increasing the aqueous solubility of the molecule or yielded derivatives with reduced activity. However, conventional drug delivery systems, such as lipidic surfactants, cyclodextrins and emulsions, failed at improving the solubility of Retro-2.1. As a result, in vivo efficacy data were obtained only with the initial hit Retro-2 so far and not its optimized analogue Retro-2.1 [5,10,14].

Recently developed drug delivery systems, such as polymeric surfactants, represent a potential alternative to these conventional systems. Indeed, polyether–block–polyester and, particularly, poly(ethylene glycol)-*block*-poly(D,L)lactide (PEG-PLA), are known to efficiently dissolve hydrophobic drugs. This phenomenon is due to the spontaneous assembly of these surfactants in aqueous solution into core–shell nanostructures known as micelles. These nano-objects possess a hydrophobic core able to host hydrophobic drugs and to shield them from the aqueous environment. The payload is then usually released in a sustained manner, despite an initial partial burst release [19]. To date, this type of system has mostly been used to encapsulate anticancer drugs. However, PEG–PLA micelles have great potential for systemic delivery of hydrophobic drugs, such as Retro-2.1 [20]. As initial results led to insufficient exposure in mice, we formulated Retro-2.1 in the chemically related PLGA–PEG–PLGA thermosensitive hydrogel. This polymer has, indeed, been described to form free-flowing solutions when dissolved in water at low temperature. Like PEG–PLA aqueous solutions, PLGA–PEG–PLGA solutions are able to dissolve hydrophobic compounds. However, upon heating, the micelles formed by PLGA–PEG–PLGA chains interact with each other, leading to the formation of a hydrogel. This phenomenon can be exploited to generate a free-flowing formulation of a hydrophobic drug candidate at room temperature that will spontaneously form an hydrogel upon warming up at body temperature (i.e., upon subcutaneous injection) [21,22,23]. Here, this strategy was applied to Retro-2.1 and led to sustained release and better control of its metabolism.

This work therefore reports the development of parenteral formulations of Retro-2.1 using PEG–PLA micelles and PLGA–PEG–PLGA thermosensitive hydrogels. These formulations allowed for the evaluation of the pharmacological properties of Retro-2.1 in vivo, starting with its pharmacokinetic parameters, as well as its metabolism. A better understanding of the fate of the drug candidate in vivo could pave the way towards the conception of a generic formulation and mode of administration to evaluate its potential in numerous in vivo models of intoxication and infectious diseases.

## 2. Results

In this study, PEG–PLA with an average molecular weight number (Mn) of 4000 g/mol (Mn(PEG) = 2000 g/mol, Mn(PLA) = 2000 g/mol) was chosen since this polymer has already been used in the past to develop Genexol^®^-PM, a paclitaxel formulation proved to be safe and efficient in a clinical setting [24,25]. Retro-2.1 was encapsulated in PEG–PLA micelles using the thin-film hydration method, also known as the solvent casting method [26]. This method allowed us to dissolve 4.55 mg/mL of the drug in 26.4 mg/mL of PEG–PLA solution, with an encapsulation yield of 91% and a drug loading of 15%, as determined with HPLC analysis using a calibration curve (Figure S1). Dynamic light scattering (DLS) analysis of the resulting formulation revealed the presence of micelles with a mean diameter of 22.5 nm (standard deviation = 17.34%, PDI = 0.23609, d_10_/d_50_/d_90_ = 18.58/22.36/26.9 nm). In comparison, empty micelles prepared under the same conditions had a mean diameter of 20.6 nm (standard deviation = 13.45%, PDI = 0.23203, d_10_/d_50_/d_90_ = 17.74/20.38/24.52 nm); see Figure S2. As previously described, including for Genexol^®^-PM, these micelles are likely to be spherical [27,28]. Although the zeta potential of these micelles was not determined in this study, PEG–PLA micelles are widely described to have a neutral to slightly negatively charged surface [29,30,31,32,33]. The formulation was found to be stable at 4 °C and −20 °C for at least 48 h and was able to withstand at least one freeze/thaw cycle without any payload leak. However, about 35% of the drug load precipitated upon storage at room temperature for 24 h (Figure S3). Every subsequent formulation was therefore stored at −20 °C and thawed at 4 °C before use.

**Figure 2:**
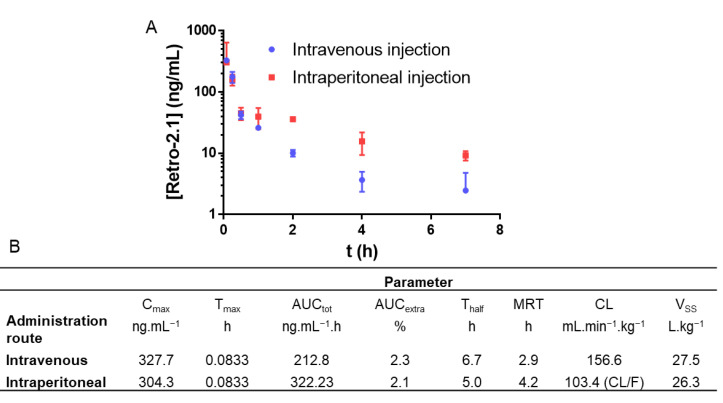
(**A**) Retro-2.1 plasmatic concentration following IV or IP injection to mice at a dose of 2 mg/kg formulated in PEG–PLA micelles (IV: *n* = 3/timepoint, IP: *n* = 5/timepoint). Results are presented as the mean, and error bars represent the standard deviation. Timepoints: 5 min, 15 min, 30 min, 1 h, 2 h, 4 h, 7 h and 24 h (not shown). (**B**) Corresponding pharmacokinetic parameters. C_max_: maximal concentration observed; T_max_: C_max_-corresponding timepoint; AUC_tot_: area under the curve, taking into account the extrapolated AUC; AUC_extra_: extrapolated portion of the AUC_tot_; T_half_: elimination half-life; MRT: mean residence time; CL: apparent total body clearance of Retro-2.1 from plasma (F: absorbed fraction); V_SS_: distribution volume at a steady state.

With this formulation in hand, a pharmacokinetic study was performed in mice using liquid chromatography coupled with tandem mass spectrometry (LC-MS/MS) as a quantitation method. As intravenous (IV) injections are closer to the eventual route of administration in a clinical setting, this route was chosen as a starting point. However, since intraperitoneal (IP) injections are closer to the reality of preclinical studies due to their simplicity, this route was also investigated. In both cases, the formulation was injected at a Retro-2.1 dose of 2 mg/kg. This dose was initially chosen to minimize any potential toxicity. In addition, the administration of 200 mg/kg of Retro-2 was sufficient to protect the mice against a lethal ricin challenge [1]. Since Retro-2.1 is about 100 times more potent than Retro-2, we hypothesized that a 2 mg/kg dose would be sufficient to achieve a relevant biological effect [2]. The plasmatic concentration of Retro-2.1 was plotted as a function of time, and the corresponding pharmacokinetic parameters were calculated using a non-compartmental model (Figure 2). Of note, no sign of pain or acute toxicity was observed in the mice over 24 h after administration of the formulation.

**Figure 3:**
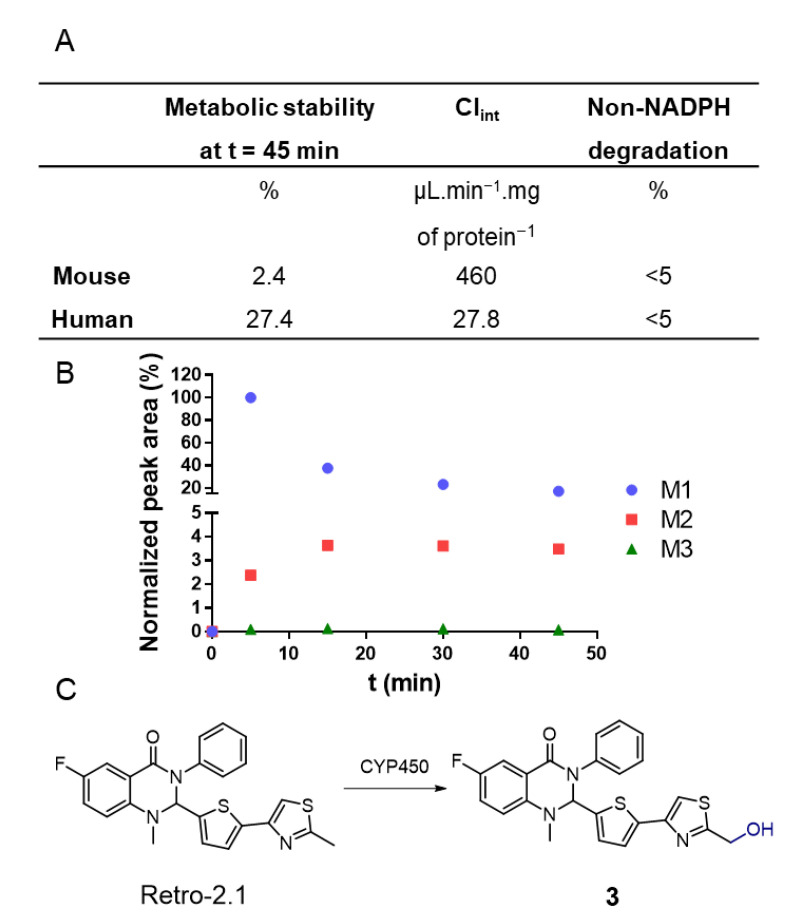
(**A**) Metabolic stability of Retro-2.1 upon incubation with mouse or human microsomes. (**B**) Normalized peak area of Retro-2.1 metabolites (M1, M2 and M3) in function of the incubation time of Retro-2.1 with mouse microsomes obtained with LC-MS. (**C**) CYP450-mediated metabolic conversion of Retro-2.1 into its major metabolite compound **3**.

Retro-2.1 was eliminated moderately quickly from the blood circulation, with elimination half-lives of 6.7 and 5.0 h for IV and IP injections, respectively. The low maximal concentrations (C_max_), areas under the curve (AUC) and high clearances are representative of unsatisfying systemic exposure with Retro-2.1. Indeed, the plasmatic concentrations of Retro-2.1 7 h following IV or IP injection were found to be 6 and 21 nM, respectively. For comparative purposes, the half maximal effective concentration (EC_50_) of Retro-2.1 against the Shiga toxin, describing the compound concentration needed to achieve 50% of its maximal protection effect, was found to be 102 nM for the racemic mixture (54 nM for the (*S*) enantiomer) [3]. One can thus infer that the plasmatic concentration achieved using this formulation and this administration route are not likely to lead to a clinically relevant exposition. Although Retro-2.1 might be eliminated by excretion, the investigation of its metabolism would help to better understand the fast elimination of the molecule from the bloodstream. This phenomenon could also lead to loss or exaltation of the drug’s biological activity or to the generation of toxic metabolites [34]. As a first step, to evaluate Retro-2.1’s metabolic stability, the drug was incubated with mouse or human hepatic microsomes, a hepatocyte fraction containing high concentrations of cytochromes P450 (CYP450), the main enzymes responsible for drug metabolism [35]. The concentration of Retro-2.1 in the samples was determined with LC-MS following 45 min of incubation (Figure 3). This preliminary study revealed that Retro-2.1 is quickly metabolized by both human and mouse microsomes. Following 45 min of incubation with mouse microsomes, only about 2% of Retro-2.1 remained in the sample, indicating particularly fast metabolism (Figure 3A). The non-NADPH degradation was found to be negligible, indicating that the metabolism is mediated by CYP450. These results potentially explain the fast disappearance of Retro-2.1 from the bloodstream following its administration in mice. To further characterize the generated metabolites, the experiment with mouse microsomes was repeated and the mass spectrum of the extracted metabolites was followed over time. The molecular mass of the metabolites was determined, and the intensity of the corresponding chromatographic peaks were plotted as a function of time (Figure 3B). Three metabolites, namely M1, M2 and M3, with respective increments of 16, 32 and 48 g/mol were detected, indicating a sequential metabolic oxidation of Retro-2.1. Given that the maximum peak area of M2 and M3 is only a fraction of the minimum peak area of M1, M2 and M3 were neglected at this stage. Of note, similar results were obtained with human microsomes, although only two metabolites with molecular weight increments of 16 and 32 g/mol were detected (Figure S4). The LC-MS/MS fragmentation data (Figure S5) obtained with the metabolite samples indicated that the oxidation takes place either on the thiophene group or on the methyl thiazole group of Retro-2.1. The LC retention time and MS/MS fragmentation pattern of the metabolite M1 were found to be identical to those of derivative **3** (Figure 3C), previously synthesized in our group during the lead optimization of Retro-2.1.

**Figure 4:**
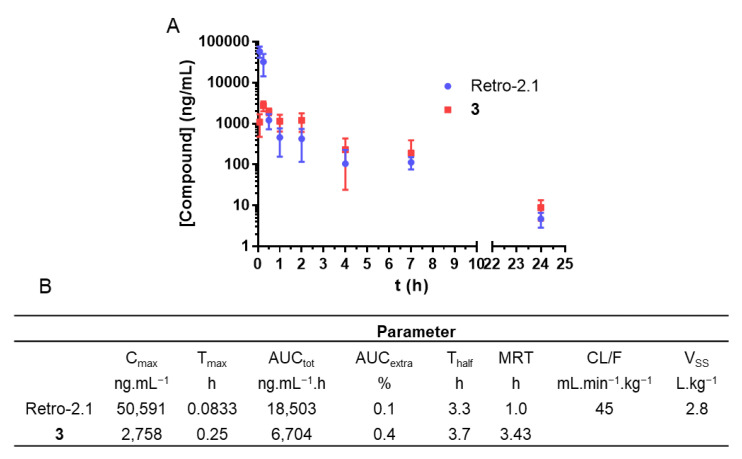
(**A**) Pharmacokinetic profile of Retro-2.1 and its major metabolite compound **3** following IP injection of 50 mg/kg of Retro-2.1 formulated in PEG–PLA (*n* = 5 mice/timepoint). Results are presented as the mean, and error bars represent the standard deviation. Timepoints: 5 min, 15 min, 30 min, 1 h, 2 h, 4 h, 7 h and 24 h. (**B**) Corresponding pharmacokinetic parameters. C_max_: maximal concentration observed; T_max_: C_max_-corresponding timepoint; AUC_tot_: area under the curve, taking into account the extrapolated AUC; AUC_extra_: extrapolated portion of the AUC_tot_; T_half_: elimination half-life; MRT: mean residence time; CL: apparent total body clearance of Retro-2.1 from plasma (F: absorbed fraction); V_SS_: distribution volume at a steady state.

The results obtained in vitro were confirmed by a second pharmacokinetic study. Retro-2.1 formulated in PEG–PLA was administered via IP injection at a dose of 50 mg/kg to mice. In contrast to the first administration dose of 2 mg/kg, and given that we did not observe any acute toxicity during the first in vivo experiment, we chose to inject 50 mg/kg of Retro-2.1 as this is the maximum dose that we could achieve, considering a recommended IP injection volume of 250 µL and the solubility of the drug candidate in this vehicle. We hypothesized that this higher dose would allow us to better detect the metabolite M1 in vivo. Blood samples were drawn at the same time intervals as previously described in this manuscript. The plasmatic concentrations of Retro-2.1 and compound **3** were determined with LC-MS/MS using an internal calibration method and plotted against time (Figure 4). Of note, no apparent sign of pain or acute toxicity was observed over 24 h following the administration of the formulation. As shown in Figure 4, Retro-2.1 is indeed metabolized into derivative **3** in mice. More importantly, only 30 min after injection, the plasmatic concentration of compound **3** exceeded that of Retro-2.1. Although the C_max_ and AUC of Retro-2.1 were considerably higher than they were previously with an injected dose of 2 mg/kg, the T_half_ and MRT remained particularly low. These results confirm that the metabolism of Retro-2.1 can partly explain its fast plasmatic elimination. At this stage, we therefore thought it important to compare the biological activities of Retro-2.1 and compound **3** in vitro. The biological activity of Retro-2.1 and compound **3** were evaluated using a Shiga-like toxin-1 (Stx-1) intoxication assay, as described previously [1,2,3]. This assay measures the ability of a test compound to protect HeLa cells against the inhibition of proteins biosynthesis induced by increasing concentrations of Stx-1. The half maximal effective concentration (EC_50_) describes the test compound concentration needed to achieve 50% of the maximal protection effect it can provide. In this assay, Retro-2.1 and compound **3** had EC_50_ values of 90 and 7381 nM, respectively. Of note, the EC_50_ of Retro-2.1 was in accordance with previously reported values [3]. These results indicate that following its IP or IV administration to mice, Retro-2.1 is quickly metabolized into an 80-fold less potent hydroxylated derivative.

This could be a significant obstacle for the preclinical evaluation of Retro-2.1. One way to improve this pharmacological profile would be to achieve sustained release of Retro-2.1, while protecting the drug from its metabolism in the liver. Subcutaneous (SC) administration of an appropriate formulation of Retro-2.1 could help to fulfil these goals.

In the late 1990s, it was discovered that aqueous solutions of the triblock copolymer poly(ethylene glycol)-*block*-poly((D,L)lactide-*co*-glycolide)-*block*-poly(ethylene glycol) (PEG–PLGA–PEG) form free-flowing solutions at room temperature and hydrogels upon heating [21]. It was later found that the copolymer PLGA–PEG–PLGA, which is more convenient to prepare, gives the same results. By tuning the composition of the polymer, it is possible to obtain an injectable solution that spontaneously solidifies at body temperature, forming an implant able to release the encapsulated drug in a sustained fashion. Such polymers are reported to be biocompatible and bioresorbable and have already proved to yield sustained release of the encapsulated drug following SC administration [21,22]. Considering the structural proximity to PEG–PLA, and the ability of the latter to dissolve Retro-2.1, we hypothesized that this polymer would be applicable to this drug candidate. Based on data from the literature, PLGA–PEG–PLGA polymer **4** was synthesized via ring-opening polymerization of a mixture of lactide and glycolide initiated by poly(ethylene glycol; Mn = 1450 g/mol) and catalyzed by tin ethyl hexanoate [23]. Polymer **4** was characterized with NMR spectrometry (Figure S6 and 7) and gel permeation chromatography (GPC, Figure S8). The main characteristics of polymer **4** are presented in Table 2.

The thin-film rehydration technique was also used to formulate Retro-2.1 in PLGA–PEG–PLGA, using acetone as a dissolution solvent and saline as a rehydration vehicle. As previously described, the polymer was prepared as a 20% (w/v) solution in saline [23]. This solution was able to stably dissolve 3.25 mg/mL of Retro-2.1, with an encapsulation yield of 98% and a drug loading of 1.9%. DLS analysis of the formulation revealed the presence of micelles with a mean diameter of 24.1 nm (standard deviation = 34.43%, PDI = 0.11852, d_10_/d_50_/d_90_ = 14.75/22.36/35.5 nm). In comparison, empty micelles had a mean diameter of 23.1 nm (standard deviation = 25.93%, PDI = 0.06724, d_10_/d_50_/d_90_ = 16.17/22.36/30.09 nm); see Figure S9. The inverted vial method showed that the formulation forms a free-flowing solution at room temperature and starts solidifying at 31 °C, which is suitable for our purpose. Of note, above 42 °C, the polymer aggregated and separated from water, which is in accordance with other reported polymers with similar structures [23]. The formulation was stable for at least 13 days when stored at 4 °C. However, like the formulation in PEG–PLA, the formulation in PLGA–PEG–PLGA lost 20% of its payload upon storage at 25 °C for 24 h (Figure S10). Subsequent formulations were therefore freshly prepared and stored at 4 °C before use.

**Figure 5:**
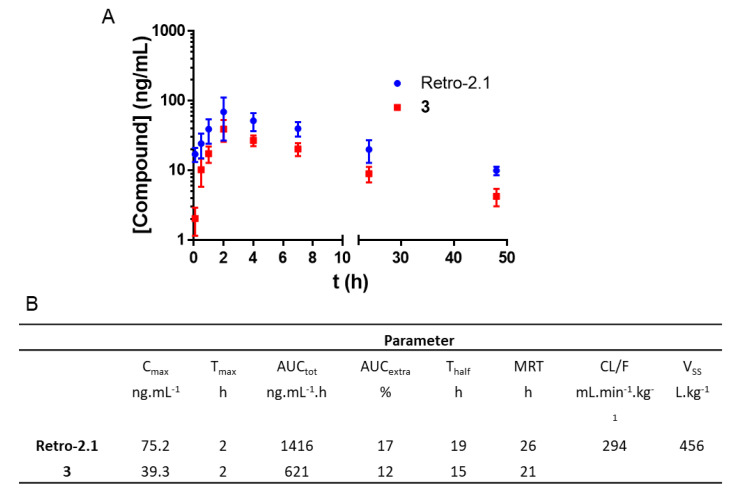
(**A**) Plasmatic concentration of Retro-2.1 and compound **3** following SC injection of 25 mg/kg of Retro-2.1 formulated in PLGA–PEG–PLGA to mice (*n* = 5/timepoint). Results are presented as the mean, and error bars represent the standard deviation. Timepoints: 5 min, 30 min, 1 h, 2 h, 4 h, 7 h, 24 h and 48 h. (**B**) Corresponding pharmacokinetic parameters. C_max_: maximal concentration observed; T_max_: C_max_-corresponding timepoint; AUC_tot_: area under the curve, taking into account the extrapolated AUC; AUC_extra_: extrapolated portion of the AUC_tot_; T_half_: elimination half-life; MRT: mean residence time; CL: apparent total body clearance of Retro-2.1 from plasma (F: absorbed fraction); V_SS_: distribution volume at a steady state.

A pharmacokinetic study was performed, as previously described in this manuscript. The PLGA–PEG–PLGA formulation was injected into mice subcutaneously, in the back, at a dose of 25 mg/kg of Retro-2.1, which is the maximum dose achievable with an injection volume of 0.15 mL. The plasmatic concentration of Retro-2.1 and its metabolite compound **3** were plotted as a function of time, and the corresponding pharmacokinetic parameters were calculated, as previously described (Figure 5). Of note, no sign of pain or acute toxicity was observed over 48 h after injection. Although the AUC was significantly lower than when Retro-2.1 was administer via IP injection at a dose of 50 mg/kg, the T_half_ in these conditions was almost six times higher (19 h). Moreover, the plasmatic concentration of Retro-2.1 remained twice superior to that of compound **3** during the whole duration of the experiment. It is worth mentioning that the pharmacokinetic parameters calculated for this route of administration must be considered with caution. Indeed, as Retro-2.1 is confined within the hydrogel, the fast elimination of the drug from the blood circulation is compensated by slow absorption from the subcutaneous compartment. This gives rise to the so-called flip-flop pharmacokinetics often encountered for drugs injected extravascularly [36]. Consequently, only an apparent elimination phase can be observed, which explains the significant difference in T_half_ between IV and SC administrations, even though the elimination of the drug should not be affected by the administration route. Still, this formulation associated with this mode of administration yielded sustained release of Retro-2.1 for at least 48 h, whereas the compound was barely detectable 24 h after intraperitoneal injection of twice the dose formulated in PEG–PLA. This mode of administration therefore provides a good alternative to the parenteral administration of Retro-2.1 formulated in PEG–PLA micelles by providing sustained release and better control of the drug metabolism.

## 3. Discussion

Where classical drug delivery vehicles failed, polymeric surfactants proved useful in improving the solubility of Retro-2.1 in aqueous media and allowed the evaluation of its pharmacokinetic and metabolic profiles following parenteral administration. Although a lot is yet to be discovered about the metabolism of Retro-2.1 (nature of the implicated CYP450 and phase II metabolism), this first study allowed us to obtain an efficient SC formulation using a PLGA–PEG–PLGA-based injectable implant. This formulation with its mode of administration unblocks the way for further studies of the pharmaceutical potential of Retro-2.1 in vivo.

## 4. Materials and Methods

### 4.1. Materials

^1^H and ^13^C NMR spectra were recorded on a Bruker Avance 400 Ultrashield at room temperature at 400 MHz and 100 MHz, respectively, and analyzed with MestRenova software. Chemical shifts were calibrated using residual undeuterated chloroform in CDCl_3_ (δ H = 7.26 ppm, δ C = 77.2 ppm) or DMSO in DMSO-*d6* (δ H = 2.50 ppm, δ H = 39.5 ppm) and were reported in parts per million (ppm), and coupling constants were reported in hertz (Hz). Splitting patterns were designed as singlet (s) or broad singlet (bs), doublet (d), triplet (t), quartet (q), quintet (quin) and multiplet (m). HPLC analyses were performed using a Shimadzu system equipped with a degasser (DEGASYS DG-1310), a system controller (Shimadzu SCL-10A VP), a binary pump system (Shimadzu LC-10AT VP) and a UV–VIS detector (Shimadzu SPD-10A VP). Compounds were separated using an ACE Excel 3 SuperC18 (100 × 4.6 mm) column at room temperature, with a flow rate of 1 mL/min (0–8.5 min: linear gradient from 100% of solvent A to 0% of solvent A from 0 to 8.5 min; 8.5–9.5 min: 0% of solvent A; solvent A: MilliQ water; solvent B: acetonitrile). Absorbance spectra at 300 nm were recorded using Borwin software. UPLC-MS analyses were performed using a Waters system equipped with a BEH Xbridge C18 (50 × 2.1 mm; 1.7 µm) column set at 40 °C (0–3.0 min: flow rate 0.4 mL/min; linear gradient from 95% of solvent A to 0% solvent A; 3.0–4.0 min: flow rate 0.6 mL/min; 0% solvent A; solvent A: MilliQ water + 0.1% HCO_2_H; solvent B: acetonitrile + 0.1% HCO_2_H), an ELSD detector (SEDEX 75, SEDERE) and a diode array detector (Acquity PDA eλ Detector) coupled to a simple quadrupole detector (SQ Detector 2). Mass spectra were recorded in both positive and negative ion modes in the *m/z* 100–2000 range and treated with Masslynx software. ESI source parameters and the gradient were as follows: capillary voltage: 3.5 kV; source temperature: 150 °C; desolvation temperature: 200 °C; cone gas flow: 20 L/h; and desolvation gas flow: 650 L/h. LC-MS/MS analyses were performed using a Waters ACQUITY UPLC^®^ system equipped with a BEH C18 (100 × 2.1 mm; 1.7 µm) column set at 50 °C (flow rate 0.6 mL/min; 0 min: 80% A; 1 min: 80% A; 18 min: linear gradient to 50% A; 18.01 min: linear gradient to 0% A; 18.50 min: 0% B; 18.51 min: linear gradient to 80% A; 25 min: 80% A; solvent A: MilliQ water + 0.1% HCO_2_H; solvent B: acetonitrile + 0.1% HCO_2_H) coupled to a XEVO TQ-S (Waters) mass spectrometer operating in positive-ion-electrospray multiple-reaction-monitoring mode. ESI source parameters were as follows: capillary voltage: 3 kV; source temperature: 150 °C; desolvation temperature: 650 °C; cone gas flow: 150 L/h; desolvation gas flow: 1200 L/h; and collision gas flow: 0.15 m/min.

### 4.2. Dynamic Light Scattering

Dynamic light scattering experiments were performed using a Vasco Flex particle size analyzer equipped with a 450 nm laser. Data were recorded at 20 °C using a refractive index of 1.331 and a solvent viscosity of 0.982 cP. For each sample, 10 acquisitions were realized using a noise limit of 1% and 200 channels with a time interval of 1 µs. All samples were analyzed at a concentration of 10 mg/mL in 4 mL glass vials (Lab File, Wheaton) unless otherwise noted.

### 4.3. Gel Permeation Chromatography

The average molecular weight number (Mn), the average molecular weight (Mw) and the polydispersity of the synthesized PLGA–PEG–PLGA were determined using a GPC 220 system from PolymerLabs (Agilent Technologies) in THF at 35 °C, with a flow rate of 1 mL/min, equipped with a series of two 7.5-mm-diameter × 300 mm Polymer Labs, 5-μm-particle-diameter mixed-E PL gel columns connected in line to the GPC system. Samples were detected using a refractive index detector. The system was calibrated using poly(ethylene glycol) or polystyrene standards (Polymer Labs) in the molecular weight range of 43580–106 g.mol^−1^. The set dn/dc value (0.050) was calculated as a weighted average of the reported dn/dc values for PEG (0.068) and PLA (0.042) [37].

### 4.4. Synthesis of poly((D,L)lactide-co-glycolide)-block-poly(ethylene glycol)-block-poly((D,L)lactide-co-glycolide) (***4***)

Adapted from a previously described procedure [23], in a flame-dried Schlenk, poly(ethylene glycol) (Mn = 1450 g/mol; 5.0 g, 3.44 mmol, 1 equiv.) was heated under vacuum at 100 °C for 2 h. (D,L)-lactide (8.6 g, 59.6 mmol, 17 equiv.) and glycolide (1.6 g, 13.8 mmol, 4 equiv.) were added to the melt under nitrogen flux. The mixture was heated at 140 °C until complete melting. Next, the mixture was dried under vacuum at 140 °C for 30 min, and Tin(II) 2-ethylhexanoate (20 mg, 0.04 mmol, 0.01 equiv.) was added. The Schlenk was purged with nitrogen, and the mixture was stirred at 140 °C for 15 h. The reaction was cooled down to room temperature, and the solid was dissolved in acetone. The organic phase was added dropwise to 100 mL of cold water. The mixture was stirred at 4 °C until complete dissolution. The aqueous phase was heated to 80 °C, and the precipitate was isolated with decantation and dissolved again in acetone. This process was repeated twice; the precipitate was dissolved in dichloromethane, dried over magnesium sulphate and filtered; and the solvent was removed under vacuum. The residue was dried under vacuum at 40 °C for 48 h to yield the desired product polymer **4** as a transparent gum (10.78 g, 2.17 mmol, 63%).

^1^H NMR (400 MHz, CDCl_3_) δ 5.28–4.98 (m, 42H), 4.93–4.48 (m, 18H), 4.42–4.16 (m, 6H), 3.62 (s, 132H), 1.62–1.41 (m, 125H).

^13^C NMR (101 MHz, CDCl_3_) δ 169.5, 166.5, 70.7, 69.3, 69.1, 16.8.

### 4.5. Preliminary Vehicle Screening

Preliminary screenings using classical drug vehicles (Tween 80 5%, Cremophor EL 5%, Solutol HS15 5%, Lutrol F68 5%, hydroxypropyl-β-cyclodextrin 10%, Crysmeb 10% or Intralipid 20%) were carried out using the CRO Drugabilis (Villejust, France).

### 4.6. Formulation of Retro-2.1 in PEG-PLA (Representative Procedure)

In a round-bottom flask, Retro-2.1 (92 mg, 211 µmol) and PEG–PLA (528 mg, 132 µmol) were dissolved in acetone (20 mL). The solvent was removed under reduced pressure to yield a transparent pale-yellow polymeric film, which was rehydrated with saline (15 mL) at 40 °C. Next, 2M NaOH solution in water was added to reach a pH of 7. The volume was adjusted to 20 mL with saline, and the solution was sterile-filtered on a 0.2 µm membrane (Millex^®^, Duluth, GA, USA) into several 6 mL sterile vials and stored at −20 °C until further use. The Retro-2.1 concentration was determined using HPLC after dilution of the solution in MeOH. Drug loadings and encapsulation efficiencies were calculated using the following formula:

(1) $$encapsulation\;yield=100*\frac{m\left(drug\;dissolved\right)}{m\left(drug\;added\right)}\;drug\;loading=100*\;\frac{m\left(drug\right)}{m\left(drug\right)+m\left(polymer\right)}$$

### 4.7. Formulation of Retro-2.1 in PLGA-PEG-PLGA (Representative Procedure)

In a round-bottom flask, Retro-2.1 (59.8 mg, 137 µmol) and PLGA–PEG–PLGA (3.00 g, 545 µmol) were dissolved in acetone (15 mL). The solvent was removed under reduced pressure to yield a translucent pale-yellow polymeric film, which was further dried under vacuum for 4 h. The film was rehydrated with saline (15 mL) at room temperature, and 2 M NaOH solution in water was added to reach a pH of 7. The solution was sterile-filtered on a 0.2 µm membrane (Millex^®^) into 6 mL sterile vials and stored at 4 °C until further use. The Retro-2.1 concentration was determined using HPLC after dilution of the solution in MeCN. Drug loadings and encapsulation efficiencies were calculated, as described before.

### 4.8. Formulations’ Stability Evaluation

A sample of the formulation was placed in a 500 µL Eppendorf tube and stored at −20 °C, 4 °C or room temperature (20–25 °C). At each timepoint, the sample was centrifuged (15,000× *g*, 5 min, 4 °C) and the Retro-2.1 concentration in the supernatant was determined using HPLC.

### 4.9. Animal Experiments

Animal care and procedures were performed according to Directive 2010/63/EU of the European Parliament, which had been approved by the Ministry of Agriculture, France. The project was submitted to the French Ethics Committee CEEA (Comité d’Ethique en Expérimentation Animale) for authorization and approved by the Minister of Higher Education and Research. Female BALB/c mice weighing 20–23 g were provided by Janvier Labs and acclimatized at least for 1 week before experiments. When indicated, the mice were anaesthetized with a ketamine (Imalgen^®^, 150 mg/kg)/xylazine (Rompun^®^, 10 mg/kg) mixture. All equipment in contact with blood was coated with heparin (Héparine Cholay; 5000 UI/mL).

### 4.10. Formulation Administration and Blood Sampling

Mice were randomly divided into 8 groups (*n* = 3 mice/group for IV injections, *n* = 5 mice/group for IP and SC administrations). Each group was assigned to a timepoint (5 min, 15 min, 30 min, 1 h, 2 h, 4 h, 7 h and 24 h or 5 min, 30 min, 1 h, 2 h, 4 h, 7 h, 24 h and 48 h for the Retro-2.1@PLGA–PEG–PLGA formulation). The mice were weighed and administered with the test formulation at t0. At each timepoint, the mice were anesthetized with a ketamine/xylazine mixture, blood was drawn by cardiac puncture with a sodium-heparin-coated needle and syringe and the animals were euthanized. Blood was transferred into a sodium-heparin-coated Eppendorf tube, which was centrifuged at 16,000× *g* for 10 min. Plasma samples were transferred to individual Eppendorf tubes and stored at −20 °C until titration.

### 4.11. Retro-2.1 Plasmatic Concentration Determination

To a mixture of 50 µL of mouse plasma and distilled water (5 µL) were added 5 µL of an internal standard 10 µg/mL solution in water and 300 µL of MeCN. The mixture was agitated at 1500 rpm at room temperature for 10 min. The mixture was then centrifuged at 20,000× *g* at 5 °C for 10 min. The supernatant was isolated and evaporated under nitrogen flux at 40 °C. The residue was dissolved in 50 µL of MeCN. The mixture was centrifuged at 20,000× *g* at 5 °C for 5 min, and the supernatant was analyzed using LC-MS/MS. The Retro-2.1 concentration was determined by comparing the intensity of the molecular ion to a calibration curve acquired under the same experimental conditions. The quantitation method was validated in accordance with the EMA Guideline on bioanalytical method validation.

### 4.12. Microsomal Stability Assay

Retro-2.1 was incubated at a concentration of 5 µM in mouse (0.5 mg/mL in PBS, MIC255, batch MIC255030, Biopredic) or human (1.0 mg/mL in PBS, MIC259, batch MIC259822, Biopredic) microsomes at 37 °C with or without NADPH as a cofactor. At each timepoint, enzymatic digestion was stopped by addition of MeCN. The mixture was centrifuged at 20,000× *g* for 20 min, and the supernatant was analyzed using LC-MS/MS.

### 4.13. Cell Culture

HeLa cells (human cervical tumour cells) were maintained in Dulbecco’s Modified Eagle’s Medium (DMEM, Invitrogen) supplemented with 1% non-essential amino acid solution (MEM NEAA, Invitrogen), 10% foetal bovine serum, 100 U/mL of penicillin and 100 µg/mL of streptomycin (referred to as complete DMEM hereafter) at 37 °C under 5% CO_2_.

### 4.14. Shiga Toxin Cell Intoxication Assay

HeLa cells suspended in complete DMEM were seeded at a density of 20,000 cells/100 µL/well in a 96-well plate with a scintillant-incorporated base (CytoStar-T 96-well plate, PerkinElmer). The plate was incubated at 37 °C under 5% CO_2_ for 24 h. The cells were incubated with increasing concentrations of the test compound by adding 50 µL/well compound dilutions in complete DMEM at 37 °C under 5% CO_2_ for 4 h. The cells were then challenged with increasing concentrations of Stx-1 (from 10^−9^ to 10^−16^ M) in the continuous presence of the test compound at 37 °C under 5% CO_2_ for 15 h. The medium was replaced with 100 µL/well of complete DMEM without leucine and supplemented with 0.5 µCi/mL of [^14^C]-leucine, and the cells were incubated at 37 °C under 5% CO_2_ for 6 h. The plate was read using a Wallac 1450 MicroBeta liquid scintillation counter (PerkinElmer). The number of counts per minute from duplicate wells was plotted against the Stx-1 concentration, and data were normalized by setting the number of counts per minute of the well containing 10^−16^ M Stx-1 to 100% protein biosynthesis. Data were fitted with Prism v5 software (Graphpad Inc., San Diego, CA, USA). IC_50_ values, corresponding to the Shiga toxin concentration required to inhibit 50% of the protein biosynthesis, were determined using non-linear regression (equation: EC_50_ shift). R was defined as the ratio between IC_50_ of the test compound at a given concentration and IC_50_ of the vehicle. *%protection* represents the protection obtained at a given concentration of the test compound compared to the maximum protection achieved in the assay and is calculated with the following formula:

(2) $$\%protection=\frac{R-1}{Rmax-1}\times 100,\;\mathrm{with}\;R=\frac{IC_{50}\left(test\;compound\right)}{IC_{50}\left(vehicule\right)}$$

The test compound concentration was plotted against the %protection, and the EC_50_ value corresponding to the test compound concentration required to achieve 50% of the maximum protection was determined using non-linear regression (equation: log(inhibitor) vs. response–variable slope).

## Figures and Tables

**Table 1:** Pathogens affected by Retro-2 and its derivatives.

Class of Pathogen	Pathogen	References
Toxins	Ricin Shiga toxins	[1,4]
Viruses	Adeno-associated virus Polyoma virus Papilloma virus Ebola virus Marburg virus Poxvirus, Vaccinia Cytomegalovirus Enterovirus 71 Herpes simplex virus type 2 SARS-CoV-2	[1,2,3,5] [6] [7,8] [8] [9] [9] [10,11] [12] [14] [13] [17]
Parasites	*Leishmania amazonensis*	[15]
Intracellular bacteria	*Simkania negevensis* *Chlamydia*	[16] [4]

**Table 2:** Synthesized PLGA–PEG–PLGA polymer characteristics. LA/GA: lactide-to-glycolide molar ratio; Mn: average molecular weight number; Mw: average molecular weight.

Mn (PEG) (g/mol)	Mn ^1^ (g/mol)	LA/GA theoretical	LA/GA Experimental	Mn^2^ (g/mol)	Mw ^2^ (g/mol)	Polydispersity ^2^
1450	4976	4.32	4.7	4205	4969	1.182

^1^ Determined with ^1^H-NMR. ^2^ Determined with GPC.

## Data Availability

Data are available from the authors upon reasonable request.

## Supplementary Materials

The following supporting information can be downloaded at https://www.mdpi.com/article/10.3390/ijms232314611/s1: Figure S1: Retro-2.1 calibration curve obtained using HPLC; Figure S2: Volume size distribution determined using DLS of PEG–PLA micelles; Figure S3: Stability of the formulation of Retro-2.1 in PEG–PLA micelles; Figure S4: Metabolic stability of Retro-2.1 on human microsomes; Figure S5: Retro-2.1 and M1 fragmentation patterns obtained using MS/MS; Figure S6: ^1^H NMR spectrum of the synthesized PLGA–PEG–PLGA in CDCl_3_; Figure S7: ^13^C NMR spectrum of the synthesized PLGA–PEG–PLGA in CDCl_3_; Figure S8: GPC chromatogram of the synthesized PLGA–PEG–PLGA in THF; Figure S9: Volume size distribution determined using DLS of PLGA–PEG–PLGA micelles; Figure S10: Stability of the formulation of Retro-2.1 in PLGA–PEG–PLGA micelles; Figure S11: Synthesis scheme of compound **3** and corresponding procedures.
