# Supplementary material for: In Vivo Sustained Release of the Retrograde Transport Inhibitor Retro-2.1 Formulated in a Thermosensitive Hydrogel
Source: Int J Mol Sci. 2022 Nov 23;23(23):14611. doi: 10.3390/ijms232314611 (PMC9735573; doi:10.3390/ijms232314611)
Supplement: Supplementary file 1 [file ijms-23-14611-s001.zip › ijms-2031312-supplementary.pdf]

Supporting Information

In vivo sustained release of the retrograde transport inhibitor Retro-2.1 formulated in a thermosensitive hydrogel

Robin Vinck [1,2], Laetitia Anvi Nguyen [3], Mathilde Munier, [2] Lucie Caramelle, [1] Diana Karpman, [4] Julien Barbier, [1] Alain Pruvost, [3] Jean-Christophe Cintrat [2,*] and Daniel Gillet [1,*]

[1] Université Paris-Saclay, CEA, INRAE, Département Médicaments et Technologies pour la Santé (DMTS), SIMoS, 91191 Gif-sur-Yvette, France

[2] Université Paris-Saclay, CEA, INRAE, Département Médicaments et Technologies pour la Santé (DMTS), SCBM, 91191 Gif-sur-Yvette, France

[3] Université Paris-Saclay, CEA, INRAE, Département Médicaments et Technologies pour la Santé (DMTS), SPI, 91191 Gif-sur-Yvette, France

[4] Department of Pediatrics, Clinical Sciences Lund, Lund University, Lund, Sweden

***** Correspondence: DG: Daniel.gillet@cea.fr; Tel. +33 (0)1 69 08 76 46. JCC: Jean-christophe.cintrat@cea.fr; Tel. +33 (0)1 69 08 21 07

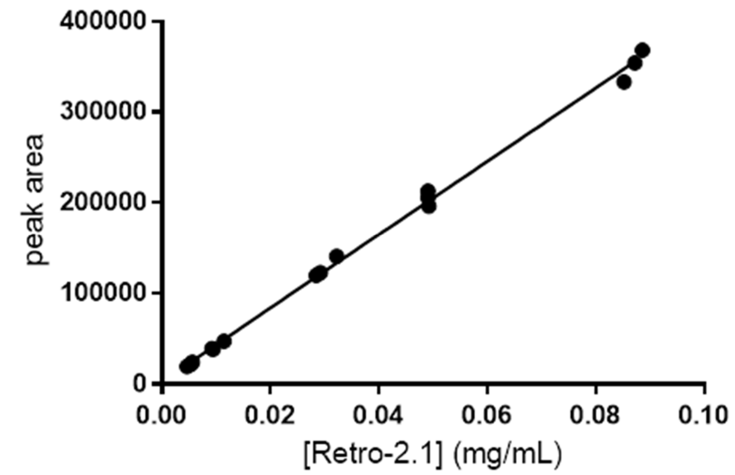

Figure S1: Retro-2.1 calibration curve obtained by HPLC. Each dilution was prepared independently by dissolving a known amount of Retro-2.1 in methanol. The chromatograms were recorded at 300 nm and the peak corresponding to Retro-2.1 was integrated.

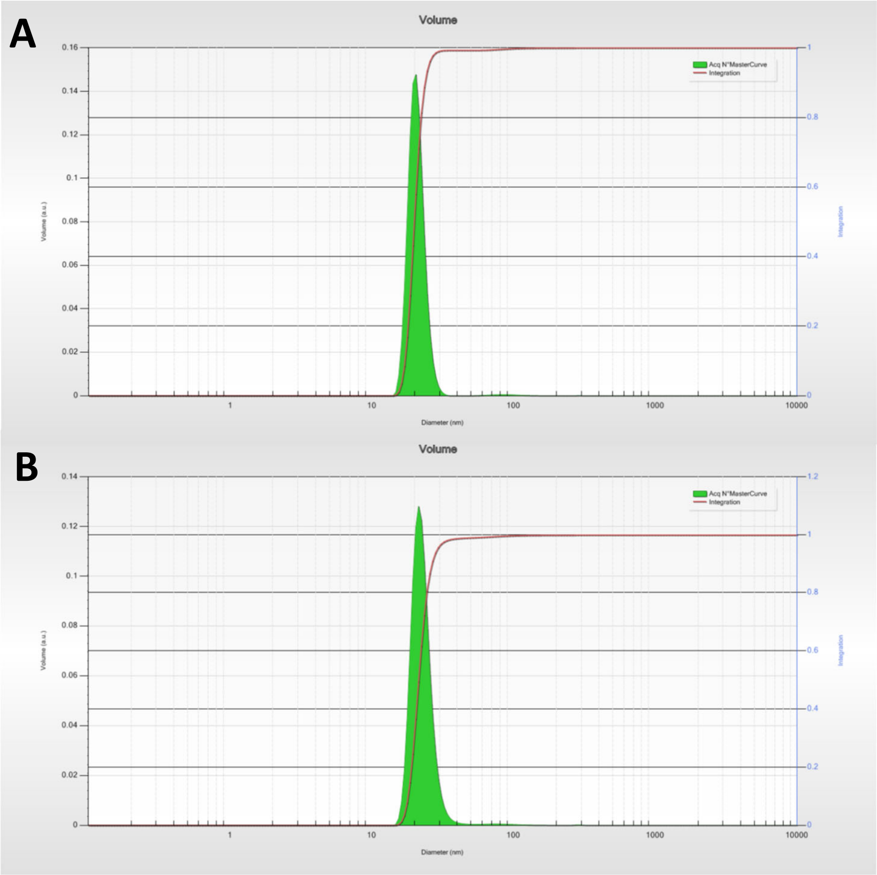

Figure S2: Volume size distribution determined by DLS of **A.** empty PEG-PLA micelles. **B.** PEG-PLA micelles containing Retro-2.1.

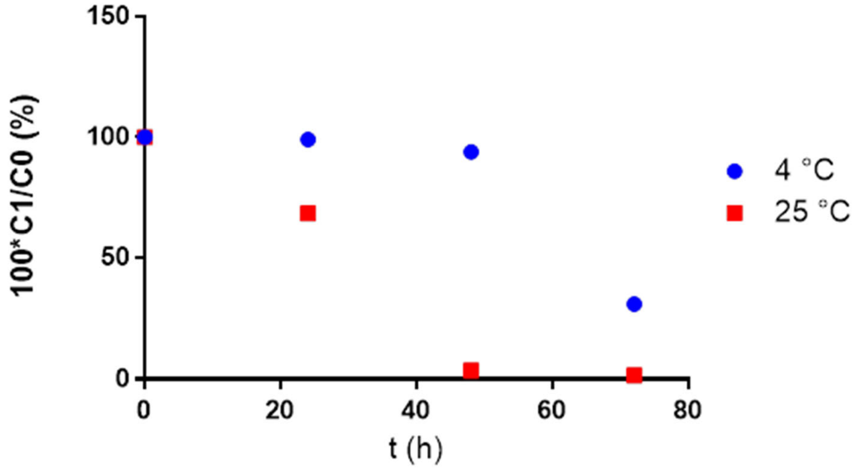

Figure S3: Stability of the formulation of Retro-2.1 in PEG-PLA micelles. The amount of Retro-2.1 in solution (C_1) was followed overtime and normalized with the initial concentration (C_0). The experiment was performed at 4 °C and at 25 °C.

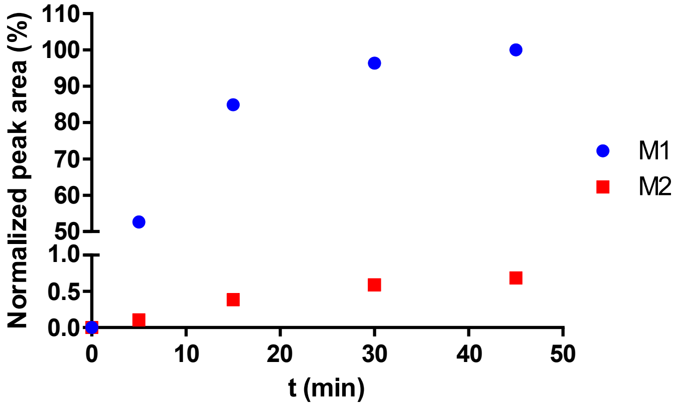

Figure S4: Metabolic stability of Retro-2.1 on human microsomes. Two metabolites M1 and M2 were detected, with molecular weight increments of 16 and 32 g/mol respectively in regard to Retro-2.1 molecular mass.

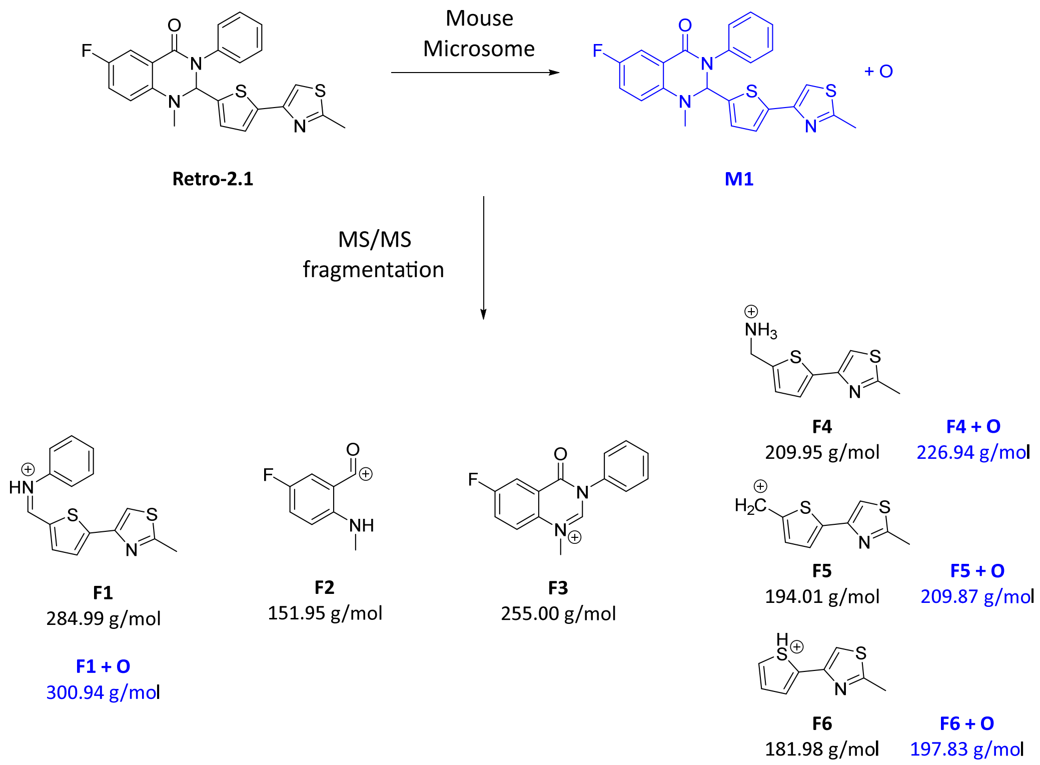

Figure S5. Retro-2.1 and M1 fragmentation patterns obtained by MS/MS. Fragments annoted in black are common to both molecules. Fragments annoted in blue were only observed for M1. For each fragment, only the experimental mass was annoted.

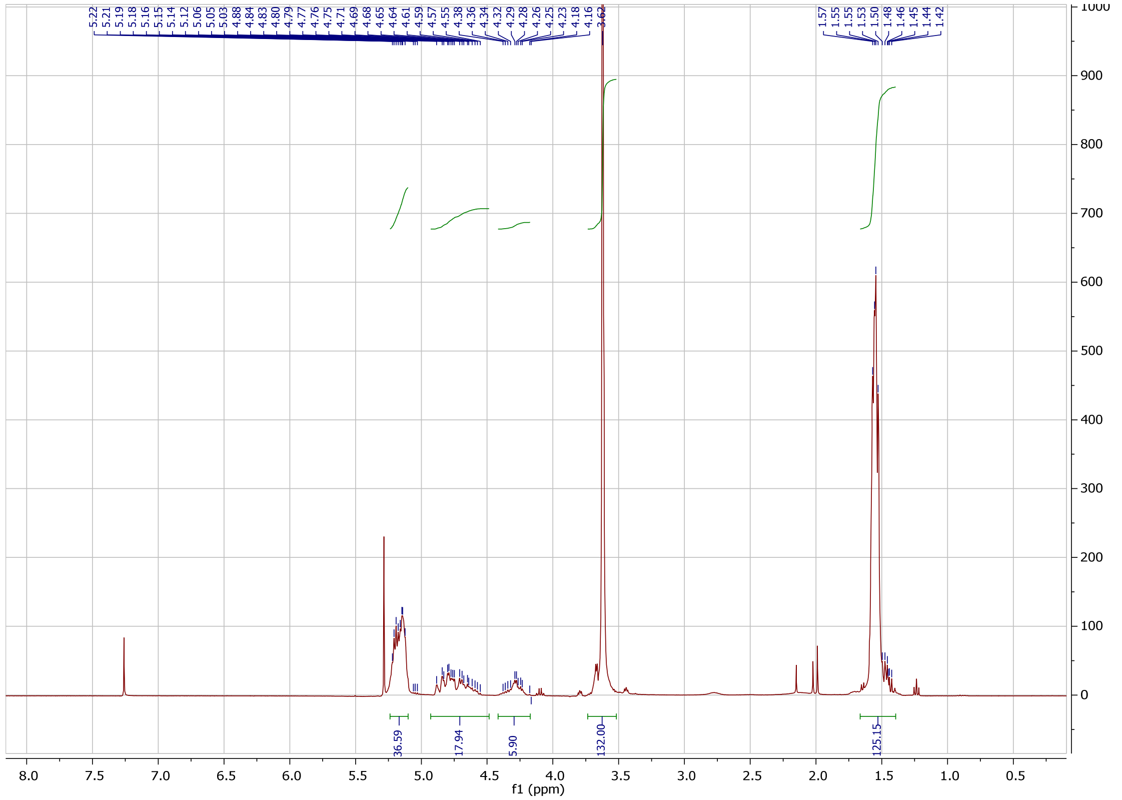

Figure S6: ^{1}H NMR spectrum of the synthesized PLGA-PEG-PLGA in $CDCl_3$

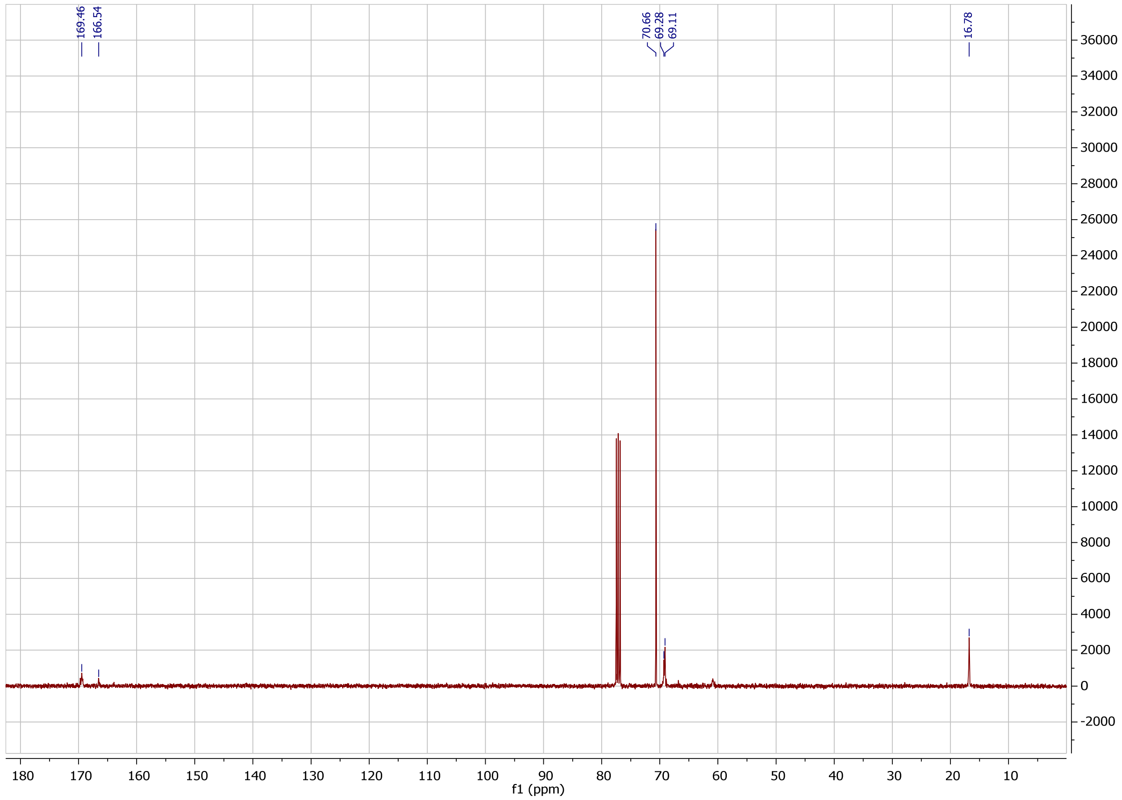

Figure S7: ^{13}C NMR spectrum of the synthesized PLGA-PEG-PLGA in $CDCl_3$.

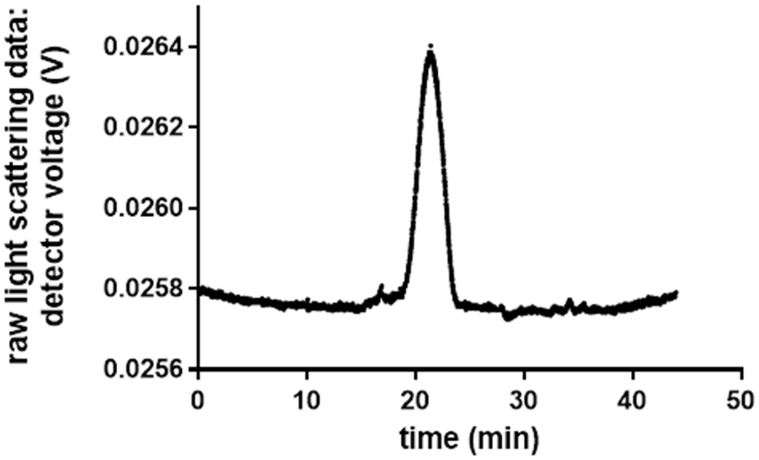

Figure S8: GPC chromatogram of the synthesized PLGA-PEG-PLGA in THF.

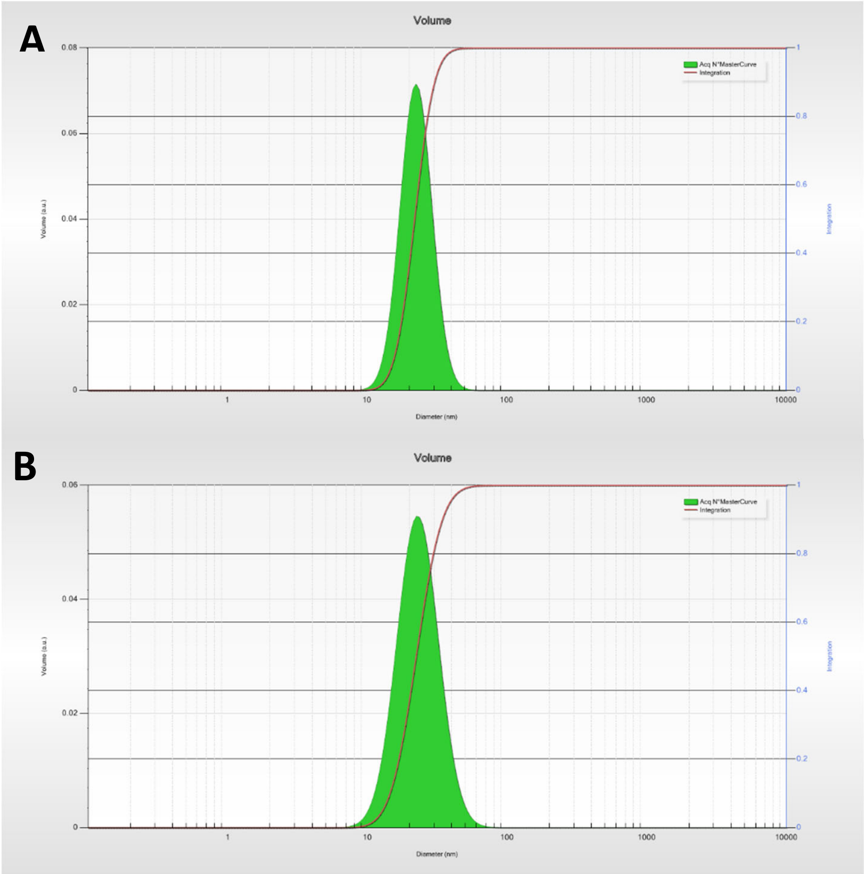

Figure S9: Volume size distribution determined by DLS of **A.** empty PLGA-PEG-PLGA micelles. **B.** PLGA-PEG-PLGA micelles containing Retro-2.1.

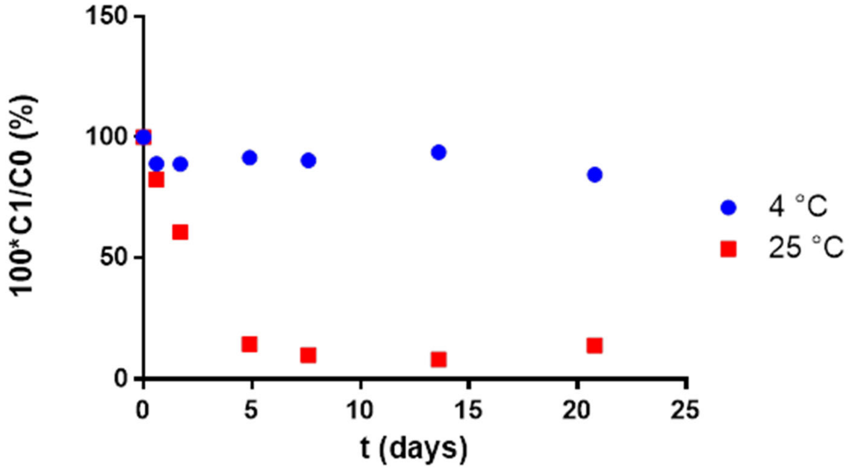

Figure S10: Stability of the formulation of Retro-2.1 in PLGA-PEG-PLGA micelles. The amount of Retro-2.1 in solution (C_1) was followed overtime and normalized with the initial concentration (C_0). The experiment was performed at 4 °C and at 25 °C.

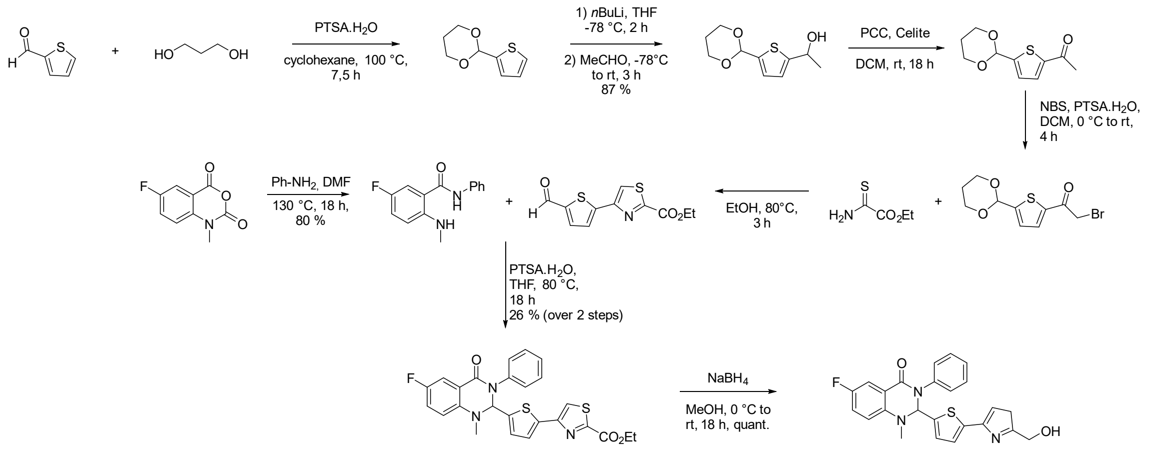

Figure S11: Synthesis scheme of compound **3** and corresponding procedures.

Detailed synthesis of compound **3**:

2-(thiophen-2-yl)-1,3-dioxane

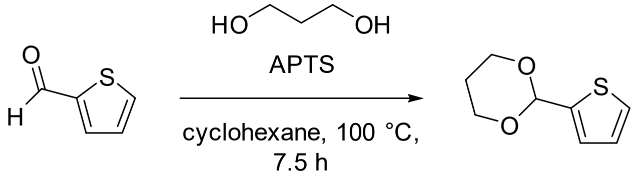

A mixture of 2-thiophenecarbaldehyde (1.5 mL, 16 mmol, 1 equiv.), PTSA monohydrate (610 mg, 3.2 mmol, 0.2 equiv.), 1,3-propandiol (2.3 mL, 32 mmol, 2 equiv.) in cyclohexane (32 mL) was refluxed in a Dean-Stark for 7.5 h. The mixture was cooled down, filtered and the organic layer was washed with a saturated solution of $NaHCO_3$ (2 x 30 mL), dried over $MgSO_4$,

filtered and evaporated under reduced pressure to give 2-(thiophen-2-yl)-1,3-dioxane (2.3 g, 13.5 mmol, 84%) as a brown oil that was used without purification. The spectroscopic data are in accordance with the literature.[33]

^{1}H NMR (400 MHz, $CDCl_3$) δ 7.29 (dd, J = 5.0, 1.2 Hz, 1H), 7.14 – 7.11 (m, 1H), 6.99 (dd, J = 5.0, 3.6 Hz, 1H), 5.75 (s, 1H), 4.33 – 4.19 (m, 2H), 4.04 – 3.91 (m, 2H), 2.29-2.17 (m, 1H), 1.47-1.42 (m, 1H).
^{13}C NMR (101 MHz, $CDCl_3$) δ 141.7, 126.6, 125.8, 125.1, 98.5, 67.5, 25.6.
LC/MS (ESI)$^+$ *m/z* : $[M+H]^+$ = 171.1 (retention time 2.51 min).

1-(5-(1,3-dioxan-2-yl)thiophen-2-yl)ethan-1-ol

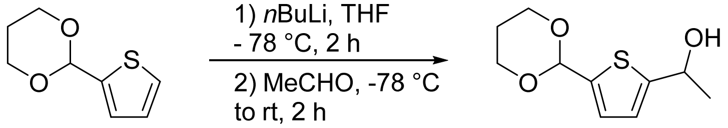

A solution of *n*-BuLi (1.95 M in cyclohexane, 2.9 mL, 1.2 equiv.) was added dropwise at -78 °C to a solution of 2-(thiophen-2-yl)-1,3-dioxane (817 mg, 4.80 mmol, 1 equiv.) in dry THF (16 mL). The resulting mixture was stirred at the same temperature for 2 h and acetaldehyde (0.805 mL, 14.1 mmol, 3 equiv) was added dropwise. The reaction mixture was warmed at room temperature and stirred for 2 h. The reaction mixture was cooled at 0 °C and quenched with a saturated solution of $NaHCO_3$ (30 mL) and the aqueous layer was extracted with EtOAc (3 x 60 mL). The combined organic layers were dried over $MgSO_4$, filtered and evaporated under reduced pressure. The crude product was purified by silica gel chromatography (cyclohexane to EtOAc/cyclohexane 4:6) to give 1-(5-(1,3-dioxan-2-yl)thiophen-2-yl)ethan-1-ol (896 mg, 4.18 mmol, 87%) as a yellow oil.
^{1}H NMR (400 MHz, $CDCl_3$) δ 6.96 (dd, J = 3.6, 0.6 Hz, 1H), 6.84 (dd, J = 3.6, 0.7 Hz, 1H), 5.69 (s, 1H), 5.06 (q, J = 6.4 Hz, 1H), 4.26-4.22 (m, 2H), 4.00-3.93 (m, 2H), 2.30 – 2.14 (m, 1H), 1.56 (d, J = 6.4 Hz, 3H), 1.46-1.41 (m, 1H).
LC/MS (ESI)$^+$ *m/z* : $[M+H]^+$ = 215.2 (retention time 2.25 min).

1-(5-(1,3-dioxan-2-yl)thiophen-2-yl)ethan-1-one

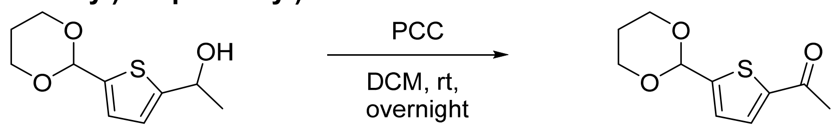

To a solution of 1-(5-(1,3-dioxan-2-yl)thiophen-2-yl)ethan-1-ol (260 mg, 1.21 mmol, 1 equiv.) in DCM (7 mL) was added in one portion PCC (415 mg, 2.06 mmol, 1.5 equiv.). The resulting mixture was stirred overnight at room temperature. The reaction was filtered on a pad of silica and washed with DCM. The solvent was removed under reduced pressure and the crude mixture was purified by silica gel chromatography (cyclohexane to EtOAc/cyclohexane 4:6) to give 1-(5-(1,3-dioxan-2-yl)thiophen-2-yl)ethan-1-one (166 mg, 0.78 mmol, 65%) as a white solid.
^{1}H NMR (400 MHz, $CDCl_3$) δ 7.60 (d, J = 3.9 Hz, 1H), 7.14 (dd, J = 3.9, 0.7 Hz, 1H), 5.73 (s, 1H), 4.35 – 4.20 (m, 2H), 4.07 – 3.92 (m, 2H), 2.55 (s, 3H), 2.29 - 2.19 (m 1H), 1.51-1.45 (m, 1H).
^{13}C NMR (101 MHz, $CDCl_3$) δ 190.9, 149.9, 144.1, 132.0, 125.7, 97.9, 67.5, 26.9, 25.6.
LC/MS (ESI)$^+$ *m/z* : $[M+H]^+$ = 213.1, $[2M+H]^+$ = 425.3 (retention time 2.44 min).

1-(5-(1,3-dioxan-2-yl)thiophen-2-yl)-2-bromoethan-1-one

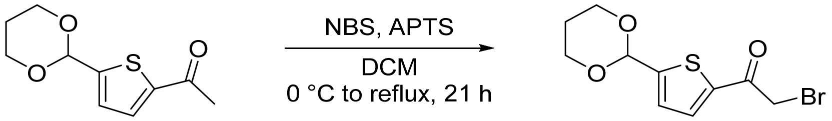

A solution of 1-(5-(1,3-dioxan-2-yl)thiophen-2-yl)ethan-1-one (160 mg, 0.75 mmol, 1 equiv.) in DCM (0.5 mL) was added at 0 °C to a solution of NBS (160 mg, 0.90 mmol, 1.2 equiv.), PTSA monohydrate (14 mg, 0.075 mmol, 0.1 equiv.) in DCM (1 mL). The resulting mixture was refluxed for 4 h. Water (5 mL) was added and the aqueous layer was extracted with DCM (2 x 5 mL). The combined organic layers were washed with a saturated solution of $NaHCO_3$ (10 mL), dried over $MgSO_4$, filtered and evaporated under reduced pressure. The crude was purified by silica gel chromatography (cyclohexane to EtOAc/cyclohexane 4:6) to give the desired compound 1-(5-(1,3-dioxan-2-yl)thiophen-2-yl)-2-bromoethan-1-one (120 mg, 0.41 mmol, 55%) as a white solid.

^{1}H NMR (400 MHz, $CDCl_3$) δ 7.71 (d, J = 3.9 Hz, 1H), 7.18 (dd, J = 3.9, 0.7 Hz, 1H), 5.74 (s, 1H), 4.35 (s, 2H), 4.33 – 4.20 (m, 2H), 4.05 – 3.95 (m, 2H), 2.34 – 2.16 (m, 1H), 1.53 – 1.43 (m, 1H).
^{13}C NMR (101 MHz, $CDCl_3$) δ 184.6, 151.6, 140.2, 133.3, 125.9, 97.7, 67.5, 30.7, 25.5.
LC/MS (ESI)$^+$ *m/z* : $[M+H]^+$ = 291.1, 293.0, 3 (retention time 2.79 min).

Ethyl 4-(5-formylthiophen-2-yl)thiazole-2-carboxylate

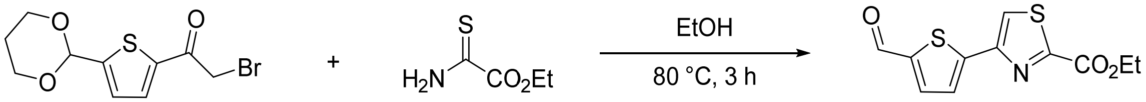

In a sealed tube, a mixture of 1-(5-(1,3-dioxan-2-yl)thiophen-2-yl)-2-bromoethan-1-one (100 mg, 0.34 mmol, 1 equiv.), ethylthioxamate (65 mg, 0.49 mmol, 1.2 equiv) in EtOH (2 mL) was heated at 80 °C for 3 h. The precipitate was filtered off and the solvent was removed under reduced pressure. The crude product was engaged in the next step without purification.

LC/MS (ESI)$^+$ *m/z* : $[M+H]^+$ = 268.1 ; $[2M+H]^+$ = 535.4 (retention time 3.01 min).

5-fluoro-2-(methylamino)-N-phenylbenzamide

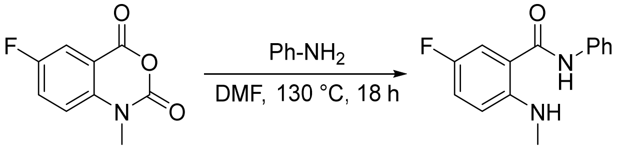

In a sealed tube, a mixture of 5-fluoro-N-methyl isatoic anhydride (500 mg, 2.56 mmol,1 equiv.), aniline (230 µL, 2.56 mmol, 1 equiv.) in DMF was heated at 130 °C for 18 h. EtOAc (5 mL) was added, and the organic layer was washed with water (5 mL). The aqueous layer was extracted with EtOAc (2 x 10 mL) and the combined organic layer were dried over $MgSO_4$, filtered and evaporated under reduced pressure. The crude product was purified by silica gel chromatography (cyclohexane to EtOAc/cyclohexane 3:7) to give the desired compound 5-fluoro-2-(methylamino)-N-phenylbenzamide (499 mg, 2.04 mmol, 80%) as a white solid. The spectroscopic data are in accordance with the literature.[3]

^{1}H NMR (400 MHz, $CDCl_3$) δ 7.71 (s, 1H), 7.58 – 7.51 (m, 2H), 7.41 – 7.33 (m, 2H), 7.23 (dd, J = 9.2, 2.9 Hz, 1H), 7.20 – 7.09 (m, 2H), 6.69 (dd, J = 9.1, 4.5 Hz, 1H), 2.86 (s, 3H).

LC/MS (ESI)$^+$ *m/z* : $[M+H]^+$ = 245.3 (retention time 3.18 min).

Ethyl 4-(5-(6-fluoro-1-methyl-4-oxo-3-phenyl-1,2,3,4-tetrahydroquinazolin-2-yl)thiophen-2-yl)thiazole-2-carboxylate

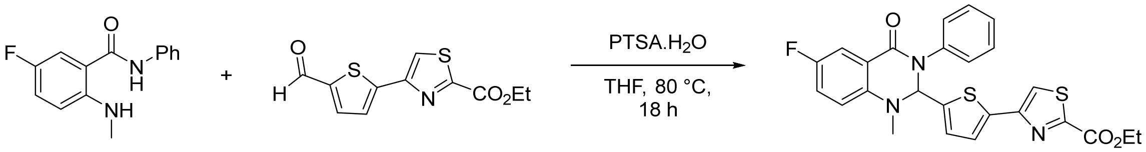

To a solution of 5-fluoro-2-(methylamino)-N-phenylbenzamide (142 mg, 0.58 mmol, 1.1 equiv.) in THF (10 mL) were added 1-(5-(1,3-dioxan-2-yl)thiophen-2-yl)-2-bromoethan-1-one (142 mg, 0.53 mmol, 1 equiv.) and PTSA.H_2O (10 mg, 0.058 mmol 0.1 equiv). The reaction was stirred at 90 °C for 18 h. EtOAc (10 mL) was added and the organic layer was washed with a saturated solution of NaHCO3 and then the aqueous layer was extracted with EtOAc (2 x 15 mL). The combined organic layer were dried over $MgSO_4$, filtered and evaporated under reduced pressure. The crude product was purified by silica gel chromatography (cyclohexane to EtOAc/cyclohexane 35:65) to give the desired compound ethyl 4-(5-(6-fluoro-1-methyl-4-oxo-3-phenyl-1,2,3,4-tetrahydroquinazolin-2-yl)thiophen-2-yl)thiazole-2-carboxylate (68 mg, 0.14 mmol, 26% over two steps) as a yellow solid.

^1H NMR (400 MHz, $CDCl_3$) δ 7.79 (dd, *J* = 8.7, 3.1 Hz, 1H), 7.51 (s, 1H), 7.42 – 7.27 (m, 6H), 7.21 – 7.10 (m, 1H), 6.85 (dd, *J* = 3.7, 0.5 Hz, 1H), 6.66 (dd, *J* = 8.9, 4.1 Hz, 1H), 5.98 (s, 1H), 4.48 (q, *J* = 7.1 Hz, 2H), 3.00 (s, 3H), 1.44 (t, *J* = 7.1 Hz, 3H).

LC/MS $(ESI)^+$ *m/z* : $[M+H]^+$ = 494.3 ; $[2M+H]^+$ = 987.7 (retention time 3.35 min).

6-fluoro-2-(5-(2-(hydroxymethyl)thiazol-4-yl)thiophen-2-yl)-1-methyl-3-phenyl-2,3-dihydroquinazolin-4(1H)-one (3)

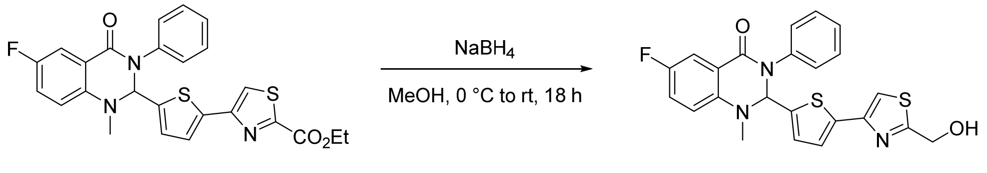

To a solution of 4-(5-(6-fluoro-1-methyl-4-oxo-3-phenyl-1,2,3,4-tetrahydroquinazolin-2-yl)thiophen-2-yl)thiazole-2-carboxylate (68 mg, 0.14 mmol, 1 equiv.) in MeOH (6.6 mL) was added at 0 °C sodium borohydride (75 mg, 2.0 mmol, 14 equiv.). The resulting mixture was stirred at room temperature for 18 h. Brine (30 mL) was added followed by a 5% HCl solution to reach pH = 7. The aqueous layer was extracted with EtOAc (3 x 50 mL). The combined organic layer were dried over $MgSO_4$, filtered and evaporated under reduced pressure to give the desired compound **3** (70 mg, 15.5 mmol, 100%) as a yellow solid.

^1H NMR (400 MHz, $CDCl_3$) δ 7.71 (dd, *J* = 8.7, 3.1 Hz, 1H), 7.36 – 7.28 (m, 2H), 7.28 – 7.17 (m, 4H), 7.13 – 7.05 (m, 2H), 6.75 (d, *J* = 3.7 Hz, 1H), 6.59 (dd, *J* = 8.9, 4.1 Hz, 1H), 5.90 (s, 1H), 4.84 (s, 2H), 2.90 (s, 3H).
LC/MS $(ESI)^+$ *m/z* : [M+H]+ = 452.3 [2M+H] = 903.5 (retention time 3.15 min)
